# Ghanaian Female Adolescents Perceived Changes in Nutritional Behaviors and Social Environment After Creating Participatory Videos: A Most Significant Change Evaluation

**DOI:** 10.1093/cdn/nzac103

**Published:** 2022-06-16

**Authors:** M Z Ghadirian, G S Marquis, N D Dodoo, N Andersson

**Affiliations:** School of Human Nutrition, McGill University, Montreal, Canada; School of Human Nutrition, McGill University, Montreal, Canada; Regional Institute for Population Studies, University of Ghana, Legon, Ghana; Department of Family Medicine, McGill University, Montreal, Canada; Centro de Investigación de Enfermedades Tropicales, Universidad Autónoma de Guerrero, Acapulco, Mexico

**Keywords:** most significant change, narrative methods, participatory research, participatory video, adolescent, nutrition literacy, rural, Ghana, nutrition education

## Abstract

**Background:**

Understanding the influence of participatory video-making on the nutrition-related behavior of video creators may help shape nutrition education interventions.

**Objectives:**

This study assessed the perceived value and influence of a participatory video intervention among participants and stakeholders.

**Methods:**

A 2018–2019 cluster randomized controlled trial (registered at clinicaltrials.gov as NCT03704649) selected 20 schools (10 intervention, *n* = 181; 10 control, *n* = 170) in 1 Ghanaian rural district, enrolled adolescent girls aged 13–16 y, and provided a nutrition curriculum. Each intervention school also participated in 2 series of activities designed to help adolescents plan, film, and screen 2 nutrition-related videos. The Most Significant Change method involved intervention participants and local stakeholders to assess the value and influence of the intervention – a secondary outcome of the trial. Project staff collected 116 stories of change from the adolescents. Stories described shifts in 4 domains: participant, peer, and family behavior, and structural changes in the school. The project team used a selection rubric to identify 14 stories that reflected heightened nutrition literacy. Staff conducted interviews with the 14 adolescents whose stories were selected to elaborate on details and perceived resonance. Finally, local stakeholders assessed the stories to identify the 4 most significant changes of the intervention – 1 per domain. A separate thematic analysis identified emerging patterns of motivation and action across the 14 interviews.

**Results:**

The chosen Most Significant Change stories revealed how adolescents found creative solutions to acquire iron-rich foods, encouraged neighbors to eat iron-rich foods, taught their family new agricultural practices, and promoted change in their school canteen. Local stakeholders valued stories that addressed common community nutrition issues in a creative and sustainable way, whereas adolescents prioritized stories that showed a change in health outcomes.

**Conclusion:**

Stories of change revealed that the intervention promoted a transformative influence; participants modified their eating habits, lifestyle, and their environment.

## Teaser

A Most Significant Change evaluation demonstrated the positive influence of a participatory video nutrition intervention on adolescent participants’ eating habits and environments.

## Introduction

Participatory videos (PVs) have been used in community development programs to amplify the voices of the marginalized, foster dialogue, educate, and advocate for change (1). This participatory approach enables people to make short films about issues affecting their lives by having control of the video-making process – identifying the topic, developing the content, acting, filming, and screening the video. Unique to these interventions is the potential 2-fold influence of: *1*) the video-making process on the thoughts and behaviors of video creators and *2*) the influence of sharing the video on the behavior of those that watch them. PV is often associated with the concept of community empowerment and social change but the influence of the PV-making process on its creators has not been the subject of rigorous research (2, 3).

PV literature often references Freire's work on critical pedagogy (4, 5) in which he called for the need for educational approaches that enhance critical consciousness (2). He posited that group reflection on images and videos may enhance participants’ critical consciousness about their own lived experiences – help them think critically about the world around them and reflect on how best to influence it (4, 5). White (6) further described how PV helps participants to become critically aware of personal and community needs, and helps them identify themselves in relation to the community which may give rise to personal, social, and cultural change.

Critical reflection is also a core concept of nutrition literacy, defined as the capacity to access and interpret nutrition information to make informed decisions (7). There are 3 levels of nutrition literacy: *1*) functional literacy, the basic skill of reading and comprehension of nutrition information; *2*) interactive literacy, the ability to apply nutrition information to new scenarios; and *3*) critical literacy, the capacity to use nutrition information to analyze one's circumstances and adjust one's personal lifestyle and influential environmental factors (8, 9). By definition, both personal and community health stand to benefit from improved critical nutrition literacy. Despite its potential benefits, the promotion of critical nutrition literacy has seldom been researched. Additionally, there is no current research on the influence of PV intervention on nutrition literacy.

Nutrition education has traditionally relied on conventional forms of social and behavior change communication such as face-to-face counseling and mass media campaigns, which do not foster community-level participation (10). An ongoing challenge for public health practitioners working in communities with low literacy is to effectively share health and nutrition information to promote behavior change (11). Participatory media, such as community radio, is becoming a valued technique for such communities to promote nutrition behavioral change (11). PV has grown in popularity as a means to promote social change, education, and health and has recently been used in nutrition-sensitive programs (11).

Research about the influence of PVs on nutrition-related behavior currently focuses on video audiences instead of video creators. In so doing, there is a lack of evidence of the effect of PV-making on the nutrition-related behavior of those who produce it, the focus of this study. This article posits that a PV education intervention could enhance critical nutrition literacy among adolescent video creators and, in turn, have an influence on the lives of participants as well as the people and environment that surround them. We seek to address another unique research gap. Little is known of the mechanisms through which nutrition literacy interventions affect participants’ behavior to promote personal and environmental change. This study aims to assess stories of change that can reveal how a nutrition literacy intervention influenced participants’ personal lifestyles and engagement in social and environmental change.

## Methods

The Most Significant Change (MSC) method is a participatory monitoring and evaluation method used to evaluate complex interventions that may have outcomes that are difficult to predict or measure (12, 13). The MSC method involves intervention participants and stakeholders in collecting and selecting stories of perceived significant change related to the intervention in question. This method complimented a quantitative evaluation that assessed the changes in adolescent participants’ nutrition literacy and lifestyles that were linked to their participation in the PV intervention. We aimed to understand the far-reaching impact of the program, a secondary outcome of the trial, by evaluating the stories of change identified by the participants. Our assumption was that, if the intervention helped adolescents improve critical nutrition literacy, then their friends, families, and surrounding environments may also be influenced. The MSC method was employed to answer the question: “What is the influence of a PV nutrition education intervention on participants’ nutrition literacy and behavior, and the perceived impact and value of the intervention to participants and other local stakeholders?” Then, an inductive thematic analysis of interviews with participants was used to answer the question: “How did the participants’ experience in the intervention influence their actions?”

### Sample

A cluster randomized controlled trial to improve the nutrition literacy of adolescent girls was carried out in 20 schools from December 2018 to August 2019 in the rural Upper Manya Krobo district, of the Eastern region of Ghana. The criteria for school selection were that they had a girls’ club, included junior high school (JHS) classes, and were within a 30-min driving radius of the district capital. Schools were randomly assigned to intervention or control arms. All schools in the study benefitted from a nutrition education curriculum. In addition, intervention participants benefitted from activities that built capacity in basic film production, discussion of nutritional issues, and development of educational nutrition videos. An analysis of the positive influence of the intervention on participants’ nutrition literacy, food knowledge, and food choices will be published elsewhere. The participant inclusion criteria were: *1*) female, aged 13–16 y, *2*) Krobo-speaking, and *3*) enrolled in a girls’ club at the time when consent and assent were acquired. Krobo-speaking field staff presented the contents of the consent and assent form in paper format (in English) and orally (in Krobo) to all guardians and adolescent participants that met the inclusion criteria in gatherings across the 20 schools. They were given ample time to ask questions. A total of 351 girls (intervention *n* = 181; control *n* = 170) met the inclusion criteria and provided informed written assent and their guardians’ informed written consent to participate. This article's MSC and qualitative analysis of participant interviews includes only the intervention schools. Data were anonymized at the point of interview transcription where participants were given an identification number. The primary investigator (MZG) was responsible for the security of identifiable data. McGill University's REB-III and the University of Ghana's Ethics Committee for the Humanities granted ethical clearance for the research to be conducted. This trial was registered at clinicaltrials.gov as NCT03704649.

### Collaborators

The MSC method involved the active participation of the participants, project team, and local stakeholders. The project team was composed of the primary investigator (PI) and project staff. The PI (MZG), a female Canadian doctoral candidate, oversaw all components of the research. The project staff were 4 local Krobo women who had experience in qualitative data collection and were responsible for implementing all intervention and data collection activities. The local stakeholders made up the Community Advisory Board (CAB) (4 men and 1 woman). They included representatives from a financial institution, a school management committee, a religious institution, Ghana Health Service, and Ghana Education Service. The CAB met monthly to refine the intervention and research methodology, participated in the research evaluation, and developed and implemented a community knowledge mobilization plan.

### Study design

In addition to attending their regular girls’ club sessions, the intervention schools (*n* = 10) participated in 2 series of PV-making activities. The first series of activities focused on helping participants (*n* = 181) produce a video on the most pressing obstacle related to acquiring a balanced diet. This process was repeated, where each intervention group produced a second video on the topic of preventing iron deficiency anemia. A total of 20 videos were made. The activities were designed to create an interactive environment for participants to reflect together on nutritional issues in their communities, to identify solutions, and to make educational videos to promote change. In the Margolis Wheel activity, adolescents were paired-off to reflect on a set of questions about the nutrition information they were taught (14). The girls rotated partners, so that they could have one-on-one conversations with different classmates. In the Problem Tree activity, they individually wrote down the most prevalent obstacle and collectively mapped out these responses into common themes. They discussed their importance and voted on what they thought was the most pressing obstacle in their community. They then drafted a storyboard that presented the pressing obstacle and their proposed solution, and acted, filmed, and screened their video once with their classmates and school staff. Intervention groups reflected on their experience within 2 wk of each screening. These group reflections provided an opportunity for participants to share observed changes they may have witnessed in themselves and their communities as a result of the intervention. The MSC approach is based on the collection of personal stories of change shared in these group reflections, an assessment of the stories by local stakeholders, and a selection of the stories of most significant change. Darts and Davies suggested a series of steps that can be adjusted to individual program aims and needs (12); the steps used for this project are described below.

### MSC steps

#### Step 1 – group discussions

The project team held a group discussion with all intervention participants at each of the 10 schools after the girls screened their first video. Here, they were asked to share observations of positive or negative changes they had seen or experienced after producing and showing the video to their peers. The project team took field notes.

#### Step 2 – domain of change identification

Domains of change were used to organize participants’ observations of significant change associated with the intervention. After reflecting on discussion field notes, the project team identified 4 domains of change: *1*) the participant's own behavior, *2*) the behavior of their peers, *3*) the behavior of their family, and *4*) their communities or schools. In addition, an ‘other’ domain was used for all other observations of change.

#### Step 3 – headline collection

The project team held a second round of group discussions with participants at each intervention school after their second videos were screened. With prompting found in **Supplemental Table 1**, participants reflected on significant changes observed in the outlined domains of change. These reflections were translated and transformed by the project team into one-sentence “headlines” that captured the main observation (e.g. “*with participant's encouragement, family starts home garden*”). A total of 116 headlines were collected.

#### Step 4 – selection rubric criteria

The project team developed a selection rubric based on the nutrition literacy levels to evaluate headlines (9). The rubric assessed the headline on 4 criteria: *1*) an understanding and application of key nutrition information, *2*) an application of information to a new or different scenario, *3*) an attempt to address an obstacle or to influence a social determinant of health, and *4*) a long-term change at the community, school, or household level. For each headline, the 4 criteria were scored as: does not demonstrate (0), somewhat demonstrates (1), or very much demonstrates (2); the final cumulative score ranged from 0 to 8.

#### Step 5 – headline selection

The project team categorized the 116 headlines into the domains of change and evaluated them using the selection rubric. Each criterion was discussed until consensus was reached. The 3 headlines with the highest score in each of the 4 defined domains were identified. Two domains had a tie and both headlines were selected. The headline selection resulted in 14 headlines. Headlines that fell into the “other” domain were statements concerning a change in health outcome and were captured by the first 4 domains.

#### Step 6 – story elaboration

The field staff conducted 14 in-depth interviews in Krobo with the participants whose headlines were selected. The interviewee was prompted (**Supplemental Table 2**) to elaborate on the details of the story, identify elements of the intervention that led to this change, and share why they thought this story was the most significant change. The interviews were translated and transcribed into English by project staff.

#### Step 7 – story writing

Two non-Ghanaian research assistants who were not previously involved in the intervention reviewed the interview transcripts and wrote a one-paragraph summary for each story. The summaries included: *1*) what the situation was like before the change occurred, *2*) what motivated the change, *3*) the sequence of events, *4*) challenges the participant faced and things that helped to facilitate change, and *5*) why this change was significant. The project team reviewed the summaries and edited as needed to assure their accuracy.

#### Step 8 – final story selection

The final selection was carried out by 3 of the CAB members; the PI facilitated the process. The 14 story summaries were read aloud by the PI, each was discussed in light of the selection rubric used previously for the headlines, and the CAB reached a consensus score for each story. The CAB members then shared why they perceived a particular story was the most significant. The PI prompted them to reflect on the differences between stories and why similar stories may have received different scores. Such probing questions allowed for rich discussion about the underlying values held by the local stakeholders. The CAB then reviewed the scores given to stories within each domain and made adjustment to scores in light of their reflections. The story with the highest cumulative score within each domain was designated the MSC story. This resulted in 4 stories.

### Secondary analysis

The PI conducted a hybrid reflexive thematic analysis of the 14 interview transcripts to further examine the participants’ perceived influence of the intervention (15). The hybrid thematic analysis included a deductive analysis of themes outlined in the semi-structured interview guide (Supplemental Table 2). The questions provided a scaffold for expected themes such as how change occurred, barriers and facilitators for change, and why participants thought their stories were the most significant. An inductive analysis identified subthemes that took shape naturally in the transcripts such as emerging patterns of action, attitudes, values, and motivation for change among participants. MaxQDA 2020 was used to conduct the thematic analysis. Results were presented to local project staff for member checking.

## Results

**FIGURE 1:**
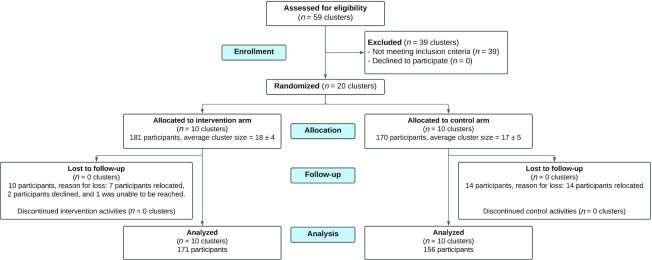
Study participant flow diagram in a cluster randomized controlled trial evaluating the effectiveness of a participatory video nutrition education intervention among adolescent girls in rural Ghana

A total of 181 adolescent girls, from the 10 intervention schools, provided informed assent and caregiver consent to participate in the trial (**Figure 1**). The average age of the 181 intervention participants was 14.3 ± 1.0 y at recruitment. A total of 16% of students (*n* = 29) were enrolled in grade 4 to 6 in primary school and 84% (*n* = 152) were enrolled in JHS in level 1 to 3. The Upper Manya Krobo district was primarily rural and underserviced. For example, only 49% of participants reported having electricity and only 2 adolescents reported having piped water in the home. The results of *1*) the final selection of stories and 2) the secondary analysis are presented here.

### Final selection of stories

**FIGURE 2:**
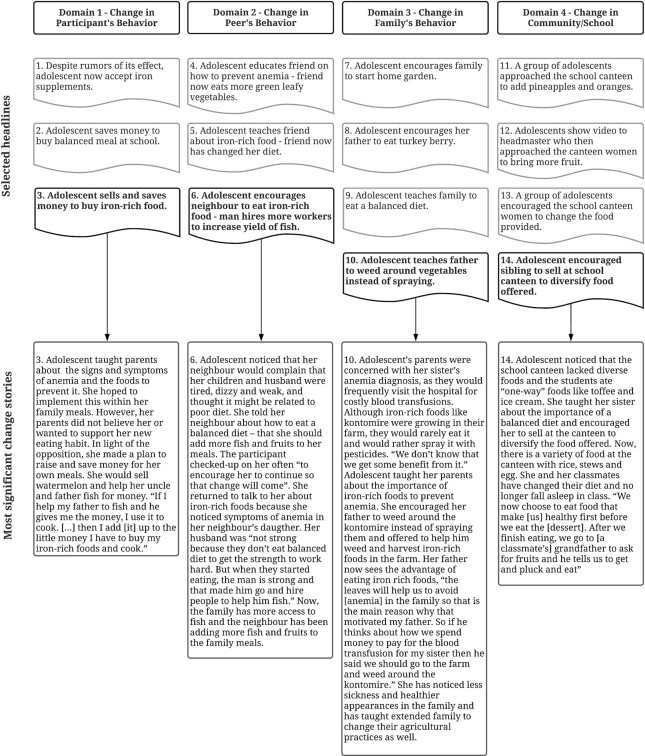
Results of a Most Significant Change evaluation of a participatory video nutrition education intervention with rural Ghanaian adolescent girls, aged 13–16 y: Headlines selected for elaboration and corresponding Most Significant Change story per domain of change

The 4 MSC stories and the reflections shared in the final selection by CAB members are described below. A summary of the MSC story per domain of change is provided in **Figure 2**.

#### Domain 1 – change in the participants’ own behavior

One story described how an adolescent accepted iron supplementation despite hearing false rumors of its adverse effects. The remaining 2 stories highlighted obstacles that the girls faced and overcame in acquiring healthy foods for themselves when family members did not accept or apply the nutrition information. The CAB members discussed the significance of the latter 2 stories, reflecting on nuanced differences in the girls’ ability to address financial obstacles in a sustainable way. Both stories highlighted the participant's efforts to acquire and save money for healthy food: 1 adolescent helped her mother sell food at the market to collect money and the second adolescent helped her father to fish as well as made efforts to sell watermelon to save money. The CAB members explained that they valued the story that exemplified the girl's determination to find creative means to generate her own income without being reliant on other people. Her perseverance in the face of obstacles, creativity, and independence showed that she understood and accepted the nutrition information. These were important reasons why this story was selected as the most significant story of change in participant behavior.

#### Domain 2 – change in peer behavior

All 3 stories outlined how the participants: *1*) identified a peer who showed symptoms of anemia, *2*) recognized its potential link to poor diet, and *3*) taught their peer about healthy eating and its benefits. The CAB valued the adolescents’ ability to persist, especially when those they were teaching had difficulty accepting the provided nutrition information. The CAB emphasized the importance of the perceived long-term effect of the story. The CAB highlighted the need for more than just changes in spending habits as this can be easily changed; 1 CAB member highlighted that *“they will take this information for some weeks, after some weeks they will reverse back”* (CAB member 1). The MSC story on peer's behavior described how the adolescent's neighbor acknowledged the importance of iron-rich foods and hired more fishermen for their business to increase their yield of fish for the family.

#### Domain 3 – change in the family behavior

Two stories depicted how the participants convinced parents of the benefits of eating balanced meals and iron-rich food. This resulted in changes in the family's spending habits, meal preparation, and consumption. The third story described how a participant encouraged her family to start a home garden in response to the perceived lack of a variety of food. The fourth story described how a participant talked to her father about changing the agricultural practice of spraying iron-rich foods like kontomire (*Xanthosoma sagittifolium*) with weedicide and offered to help weed around them instead. There was an initial tie among the latter 2 stories. To break the tie, the CAB emphasized the need to contextualize the stories in order to judge their merit. What ensued was a discussion about the underlying issues related to local agricultural practices. The CAB members reflected on discussions they have had with community members, and common agricultural practices they have witnessed to identify whether the lack of home gardens or the practice of spraying was the most prevalent issue. The CAB acknowledged that it would be difficult to convince the community to sustain home gardens despite its benefits *“what (the community) understand(s) is that the kontomire is still on their farms, so why will they make a garden? So, whether they have a garden or not, still we have those vegetables (in the farm) and we have to make use of them. Now the one challenge is that there is method of spraying – you are spraying the vegetables”* (CAB member 2). The value of the story was finally determined by reflecting on the implications of the story becoming common practice. The MSC story in family behavior was the newly adopted practice of weeding rather than spraying iron-rich foods. It addressed the most prevalent local agricultural issue and would have the greatest impact on others – if they too would adopt this behavior.

#### Domain 4 – change in community or school

All 4 stories described an increase in fruits or iron-rich foods provided in the participants’ school canteens. Due to the similarities in the story outcomes, the CAB evaluated the means taken to promote change. In 3 stories, participants approached current or potential canteen women to teach them about the need to diversify the food offered. In the fourth story, participants showed their video about the need for balanced meals at the canteen to their headmaster, who then approached the canteen women himself. The CAB saw merit in the participants’ ability to draw on influential people to help generate change. One CAB member described that this approach was wise because *“normally the teachers and the head have influence on the canteen”* (CAB member 1). Others argued that change would be more sustainable when the canteen women were taught the nutrition information themselves, saying that *“this will go a long way, because the canteen women have (understood) the whole thing”* (CAB member 3).

All agreed that the change in food provided in the canteens was praiseworthy as it increased the supply of healthy options. To determine the most sustainable approach, focus shifted towards stories that showed changes in the demand for healthier canteen food. They reasoned that if the students themselves understood and accepted the nutritional information about a balanced diet, there would be a long-term demand for healthier options at the canteens which would drive the supply. They argued that although some stories described a larger change in the types of foods provided at their canteens, the students themselves did not appear to understand the nutrition information and were rather profiting from the changes in the canteen without understanding the benefits. They claimed that although the adolescents would benefit indirectly, *“they should have the information, (and) know that (…) what I'm doing (is) right”* (CAB member 2). The MSC story was the story that demonstrated that the students themselves understood the nutritional information and that this was manifested in an increased demand for fruits instead of sweets at the canteen.

### Thematic analysis results

The following are the results of the inductive thematic analysis of the interviews with adolescent girls whose headlines were selected. Interviews explored adolescents’ patterns in motivations to spearhead change, their capacities for action, the barriers and facilitators to change, and why they deemed their story to be the most significant.

### Motivation

Adolescents were primarily motivated to influence health outcomes. Of common concern was the ill health they and their peers faced – anemia, menstrual pain and irregular flow, frequent sickness, fatigue, feeling “dull,” and being inactive. One participant explained that *“what motivated me is that my sisters always feel sick (…) so I was happy to help my father to weed around the kontomire so that we get the leaves and eat always to avoid the shortage of blood [anemia] in our family”* (15 y old, JHS level 1). Aside from the desire to address poor health, many adolescents described a motivation to be healthier, grow faster, and to look “fat” with “sparkly” skin – *“what motivated me was that when we eat such foods, we will be fat and we will be healthy. That motivated me to bring the change to the house”* (16 y old, JHS level 2).

A prominent motivator was the desire to test the nutrition information for themselves. Stories of change often started by adolescents critically appraising the validity of the nutritional claims and judging its value. For example, 1 adolescent said *“the first time that I received this education I thought it's not anything serious because my grandmother used to eat those things. So, the first thing I do is to practice it myself to see whether it is true or not”* (13 y old, JHS level 1). Another great motivation was a concern for the well-being of others and a sense of responsibility to share their newfound knowledge. One adolescent explained that *“I learned that what we learn we should not keep it to ourselves alone but we should teach others to help bring change for all of us”* (16 y old, JHS level 2).

### Capacities for action

The stories of change demonstrated that the adolescents often took a 3-step approach: *1*) identify the problem, *2*) practice the nutrition education themselves and evaluate its validity, and *3*) make a game plan to teach others. Interviewed adolescents demonstrated the capacity to identify symptoms of ill health and link them to poor diet. Many shared how they initially practiced the nutrition information for themselves to generate proof and to *“(see) if it was good”* (14 y old, JHS level 1). Participants shared how they needed to be thoughtful in the way that they taught their parents and those in higher authority like school staff. What the adolescents commonly communicated to their respective audiences – peers, family members, neighbors, and school staff – was their understanding of the nutrition information they were taught, why they thought it was important to adopt new dietary habits, and their assessment of the important issues they faced.

### Barriers and facilitators to change

The most common challenge was resistance adolescents met from parents and other elders to the nutrition information they were teaching. Other obstacles included overcoming shyness and a lack of resources (e.g., seeds, money) needed to practice what was taught. The adolescents were able to overcome obstacles and create change by identifying their strengths such as building on relationships of trust they had in their families and communities, an important facilitator. An adolescent explained how her sibling's encouragement and support motivated her to persevere in approaching their father, *“the day you came to educate us on it and I went home; I went (to) organize my younger siblings who are in the primary (school) and I made them sit as we used to sit down. I became the teacher and started teaching them on things to eat. I told one of (my sisters that) my father doesn't want to listen to me when I ask him to eat turkey berry and she encouraged me to say it more. I also continue to say it. Now if we want to cook; he will ask us to add it to the food”* (15 y old, JHS level 2). The adolescents were able to promote change because the people that they were teaching trusted them and their intentions. An adolescent shared how her neighbor told her that *“when (my neighbor) went to the hospital, she was told to eat these things, but she didn't believe, but because I have come to tell her, she believes me and she will do as I say”* (16 y old, JHS level 1).

They were encouraged to continue teaching others because of their confidence in the validity of the nutrition information and its potential to stimulate change, the social support and encouragement they received from their peers and parents, and the adoption of new behaviors around them. An adolescent shared how she and other intervention participants supported each other in promoting change. She described how, *“when they came to teach us, we all went to practice and saw how it was. When we came to the school I told (my friends that) I went to practice it and it is good and I have seen a change. (My friend) also came to tell us she has seen a change. (Two other friends) came to tell us the same thing then we said since (other students) haven't practiced it yet; we will go and talk to the canteen women so that they change the food.”* (14 y old, JHS level 1).

### Perceived significance

Adolescent interviewees were asked to share their reflections as to why their respective stories were the most significant. The common response was to judge the merit of the story by reflecting on the health outcomes seen in themselves, families, and peers. It was often argued that the story was significant because they had observed more regular menstrual flow, less menstrual pain, felt healthier and more active, or better retained what was taught in class. One participant explained, *“the important change story was that none of my family members fall sick again, no more shortage of blood (anemia) in the family again and everyone is healthy in the family, that is the most important story for me in this intervention”* (15 y old, JHS level 1). Appearance was also a valued indicator of change. They prided themselves in their ability to gain weight and have radiant skin.

### Intervention activities attributed to change

Adolescent interviewees were asked to identify the aspects of the intervention that had the most influence on change. Some referred to the new nutrition information they learned through the lessons. The process of discussing with their peers about the topic and content of the videos they were producing was particularly important to change as it facilitated understanding and a desire to teach. What stood out to the youth was the moments where they were able to have meaningful conversations with their peers in preparation for the educational video. Many said that the Margolis Wheel activity made them: *1*) feel comfortable to ask questions, *2*) retain the nutrition information they were taught, 3) comprehend new components of the lesson they previously did not understand, *4*) discuss its application in their lives, and *5*) consult and identify the most pressing obstacles. An adolescent highlighted how, *“there are some things that we don't know and if (the teacher) asks questions (…) we (feel) shy to ask, but with (this activity) we can ask our friends and we can open up more to discuss with our friends”* (13 y old, JHS level 1). They explained how these discussions helped them understand the material and build confidence to teach others. One adolescent said, *“it made me become a teacher to teach others. I like that”* (15 y old, JHS level 2) and another highlighted that *“it help(s) us to understand the lesson because we explain it to our friends”* (13 y old, JHS level 1). Few adolescents referenced the act of filming and sharing of the video as being the most important activity that motivated change.

## Discussion

This study presented evidence that PV nutrition education promoted a transformative change; participants modified their eating habits, lifestyle, and their environment. This is the first study to evaluate: *1*) the influence of a PV nutrition education intervention on adolescent participants’ critical nutrition literacy and behavior, and *2*) the perceived impact of the intervention on participants and local stakeholders.  It is also the first study of a PV nutrition intervention with adolescent creators.

Though PV has been practiced in community development since the 1960s, it has only been regularly used in nutrition promotion by SPRING since 2012 (11). Research on the use of PV as an approach for nutrition promotion is fairly new. Published studies focus on cultural acceptability and effectiveness of information uptake and personal behavior change when nutrition-sensitive information is shared through a PV medium. For example, a mixed-methods study in India showed how PVs were widely accepted and became an important source of nutrition information in communities, and increased handwashing and responsive feeding among PV audiences (16). Another mixed-methods study was conducted in Niger and Burkina Faso that promoted maternal, infant, and young child nutrition and hygiene practices through PV screenings in community gatherings (17, 18). The researchers reported that watching the PVs increased knowledge, self-efficacy, and behavior changes related to handwashing and feeding practices (17, 18). Nutrition research has focused on the audience that watches the PVs rather than those who produced them – this is where our study differs.

There was no published research highlighting the impact of participating in the PV-making process on nutrition behavior change, the focus of this study. The results of 2 studies showed the important role of participatory activities in amplifying the influence of PVs. Cai et al. (19) looked at the influence of locally made nutrition videos on audience members’ knowledge and behavior in comparison to a more participatory live food demonstration. Their results showed that although watching a PV increased knowledge, it did not improve change in nutrition behavior as much as live demonstrations (19).  Kadiyala et al. (20) evaluated 3 nutrition-sensitive agriculture (NSA) interventions in a 4-arm cluster randomized controlled trial in rural India on maternal and child dietary outcomes. The intervention arms included women's group meetings as well as: 1) NSA videos (AGRI), *2*) NSA and nutrition-specific videos (AGRI-NUT), and *3*) NSA and nutrition-specific videos, and participatory activities (AGRI-NUT + PLA).  The 2 groups that included nutrition-specific videos showed a significant difference in meeting child minimum diet diversity and maternal diet diversity (19% and 27%, respectively) compared with control groups (20).  The intervention arm with participatory activities was most influential on participant behavior, suggesting that the participatory nature of this intervention arm may be an important element that can help amplify the influence of a PV intervention (20). Further research is thus necessary to better understand the influence of PV interventions that emphasize active participation.

The concept of critical nutrition literacy is associated with cognitive and social skills to: *1*) think critically about the merit and application of nutrition information and to implement change in personal nutrition behavior and lifestyle, and *2*) to exercise one's agency to address obstacles to behavior change through increased engagement in social action (8). Our results are consistent with research highlighting the influence of PV on participants’ personal behavior – the first component of critical nutrition literacy (16, 17, 18, 19, 20, 21). There are no published studies on the influence of a PV nutrition intervention on participants’ engagement in social action – the second component of critical nutrition literacy. There were, however, 2 studies in rural India that monitored how PV audiences taught others about nutrition messages promoted in the videos (16, 21). Though the researchers detected a nascent capacity among PV audiences to teach others, they concluded that information sharing was limited (21). The focus of these studies was the degree of diffusion of the message; they did not monitor the resulting change in behavior among their social networks. Our study contributes unique evidence on the capacities of adolescents to diffuse learned messages and contribute to behavior and structural changes in their communities. Although there is little research on this topic, Kadiyala et al. (21) acknowledge that “*the ultimate impact of the intervention will depend on the effectiveness of group members to diffuse key messages to stimulate social change*.”  This article begins to contribute to this particular branch of PV nutrition education research – one that looks at the influence of nutrition PVs on social action and environmental change – and opens avenues for new research questions and explorations.

### Intervention activities attributed to change

The aim of this study was to investigate the influence and value of the PV-making process. This process involved a series of activities: peer discussion to develop the content of the videos, filming, screening videos, and reflecting with their peers about their efforts to bring about change. The interviewees did not refer to the act of filming as the most influential intervention activity that promoted change, rather they highlighted the Margolis Wheel and Problem Tree activities. These activities were foundational in developing the content of the videos. They were designed to help adolescents reflect together on the application of the nutrition information as well as the social and environmental factors that might impede its implementation. Their positive feedback to these exercises supports the premise that adolescents respond well to the opportunity to think critically about factors that affect their lives and the lives of their communities, and to propose creative solutions. These 2 activities were done within the context of a PV intervention – where the adolescents were aware that they were participating in a series of activities that would help them produce an educational video for the purpose of promoting change. Our study would not be able to suggest that these 2 activities in isolation would produce the same transformative results – additional research is needed to clarify that claim.

### Perceived value of the intervention

The MSC method helped us examine the influence and value of the intervention from the perspective of the participants and local stakeholders. This method revealed differences in the value systems of the adolescents and local stakeholders who judged the merit of stories. The interviewed adolescents commonly expressed that a positive health outcome was the reason why a story was the most significant. In contrast, the CAB valued how change occurred, the manner in which a change was put into place, the relevance of the issues it tackled, the capacities manifested, and its perceived sustainability. Though these values are not mutually exclusive, it does reflect a difference in value systems between youth and the adult CAB members. Using an MSC method, which included the participation of both youth and adults, strengthened the evaluation of the intervention because it incorporated multiple perspectives. Additionally, the difference in value systems reinforces the importance of having youth's point of view adequately represented in research relevant to their lives (3). The voice of youth can be incorporated in simple ways. A selection rubric can be developed with a wider range of local collaborators which includes youth participants. Alternatively, future projects can ensure that youth are invited to be active members of the CAB such that they have more ownership in the design, implementation, and evaluation of interventions.

### Limitations

Despite the benefits of the MSC method, there are limitations due to elements of its implementation. In this application of MSC, headlines were collected at 1 time point therefore changes over time were not captured. The headlines were also collected soon after the screening of their second video therefore, more long-term changes were not recorded. Ideally, headlines would be collected at >1 occasion with ample time in between to capture a range of changes. The nature of the MSC method is to seek out and record stories of change, whether positive or negative, giving rise to a results bias (22). Another element of the MSC method is the limited generalizability of its results. Its results help us appreciate the nuanced values and influence of the intervention in its unique communities and context.

This study contributes to the burgeoning body of research on the positive influence of PV nutrition education on knowledge and behavior. Research has focused on the influence of sharing nutrition information through a PV medium. This study, however, highlights the transformative influence of the PV-making process on addressing local nutrition issues, and empowering adolescent participants to change their own dietary habits and the environmental factors that support healthy lifestyles. Adolescents identified the PV activities that encouraged participatory peer reflection as an essential component of the intervention that influenced change. The MSC approach facilitated the evaluation of this complex behavior change intervention, as it highlighted the important personal and societal changes that resonated with adolescent participants and local stakeholders. Interventions that engage youth in the design and implementation of research facilitate the empowerment of adolescents and contribute to a myriad of changes. What is needed are creative means to involve adolescents in the design, implementation, and evaluation of programs that are meant to serve them.

## Supplementary Material

nzac103_Supplemental_File

## Data Availability

Data described in the manuscript, code book, and analytic code will be made available upon request pending application to the corresponding author (MZG) and approval.

## Notes

This work was supported by: The Canadian Queen Elizabeth II Diamond Jubilee Scholarships (QES) which is managed through a unique partnership of Universities Canada, Rideau Hall Foundation (RHF), Community Foundations of Canada (CFC), and Canadian universities. The QES-Advanced Scholars Program is made possible with financial support from the International Development Research Centre (IDRC) and Social Sciences and Humanities Research Council (SSHRC).

Author disclosures: The authors report no conflicts of interest.

Supplemental Table 1 and 2 are available from the 'Supplementary data' link in the online posting of the article and from the same link in the online table of contents at https://academic.oup.com/cdn/.

Abbreviations used: CAB, community advisory board; JHS, junior high school; MSC, Most Significant Change; NSA, nutrition-sensitive agriculture; PI, primary investigator; PV, participatory video.
