# Supplementary material for: Ghanaian Female Adolescents Perceived Changes in Nutritional Behaviors and Social Environment After Creating Participatory Videos: A Most Significant Change Evaluation
Source: Curr Dev Nutr. 2022 Jun 16;6(8):nzac103. doi: 10.1093/cdn/nzac103 (PMC9429968; doi:10.1093/cdn/nzac103)
Supplement: nzac103_Supplemental_File [file nzac103_supplemental_file.docx]

| **Supplemental Table 1**. Most Significant Change method – group discussion questions for headline collection | |
| --- | --- |
| **Questions asked to group of participants** | **Purpose of question** |
| What positive or negative changes have you observed since our last video screening? | To record a range of positive and negative changes observed by the participants without any prompts. |
| What is the most significant change you have seen in your own behaviour, if any? | To record what the participants deemed the most significant change in each of the domains of change. To identify their perception of the most significant change in 1) their own individual behaviour, 2) their peers' behaviour, 3) their family's behaviour, 4) the community, school, and surrounding environment. |
| What is the most significant change you have seen in the behaviour of your peers, if any? |  |
| What is the most significant change you have seen in the behaviour of your families, if any? |  |
| What is the most significant change you have seen in your schools or communities, if any? |  |
| Are there any other significant changes you have observed as a result of your participation in this intervention? | To record any additional significant changes experienced. |

| **Supplemental Table 2**. Most Significant Change method – in-depth interview questions | |
| --- | --- |
| **Questions asked to adolescent participant of selected headline** | **Purpose of question** |
| You had mentioned an observation about *(refer to their selected headline)* during our group reflection. Is this correct? Can you tell me more about that story? | To give the participant the opportunity to speak about the story unprompted. To give opportunity for the interviewee to mention elements of the story that seemed worth sharing. |
| What was it like before the change occurred? | To understand circumstances prior to change occurring. |
| What were the different steps that helped the change occur? | To understand how change occurred. |
| What motivated you to make this change? What do you think started this change? | To identify why the change occurred. |
| What helped you put the things you learned into practice? Was there anything hard about putting this into practice? If so, what? What helped you get over this challenge? | To identify what facilitators or barriers the participant faced in order to incite change. |
| What does it look like now? | To better understand the magnitude of change. |
| Why do you think this is the most significant change story? | To understand what changes are the most highly valued and why. |
